# Identification of sequence-specific interactions of the CD44-intracellular domain with RUNX2 in the transcription of matrix metalloprotease-9 in human prostate cancer cells

**DOI:** 10.20517/cdr.2020.21

**Published:** 2020-08-21

**Authors:** Linda T. Senbanjo, Hanan AlJohani, Mohammed AlQranei, Sunipa Majumdar, Tao Ma, Meenakshi A. Chellaiah

**Affiliations:** Department of Oncology and Diagnostic Sciences, University of Maryland School of Dentistry, Baltimore, MD 21201, USA.

**Keywords:** Prostate cancer, metastasis, CD44, RUNX2, CD44-ICD, MMP-9, tumorigenesis

## Abstract

**Aim**: The Cluster of differentiation 44 (CD44) transmembrane protein is cleaved by γ-secretase, the inhibition of which blocks CD44 cleavage. This study aimed to determine the biological consequence of CD44 cleavage and its potential interaction with Runt-related transcription factor (RUNX2) in a sequence-specific manner in PC3 prostate cancer cells.

**Methods**: Using full-length and C-terminal deletion constructs of CD44-ICD (D1-D5) expressed as stable green fluorescent protein-fusion proteins in PC3 cells, we located possible RUNX2-binding sequences.

**Results**: Chromatin immunoprecipitation assays demonstrated that the C-terminal amino acid residues between amino acids 671 and 706 in D1 to D3 constructs were indispensable for sequence-specific binding of RUNX2. This binding was minimal for sequences in the D4 and D5 constructs. Correspondingly, an increase in matrix metalloprotease-9 (MMP-9) expression was observed at the mRNA and protein levels in PC3 cells stably expressing D1–D3 constructs.

**Conclusion**: These results provide biochemical evidence for the possible sequence-specific CD44-ICD/RUNX2 interaction and its functional relationship to MMP-9 transcription in the promoter region.

## Introduction

After lung cancer, prostate cancer is the second leading cause of death in men^[1,2]^. Although treatment options for early-stage prostate cancer are beneficial, metastatic prostate cancer treatment options are more challenging^[3]^. Metastases of prostate cancer to distant sites, including bone, liver, lungs, lymph nodes, and adrenals, are often difficult to treat^[4]^. The primary treatment option for men with advanced-stage prostate cancer is androgen deprivation therapy (ADT). Although initially responsive to ADT, patients with prostate cancer eventually progress to a castration-resistant state^[5]^. Many factors contribute to prostate cancer progression and metastasis.

Cluster of differentiation 44 (CD44) is a multifunctional cell surface receptor that has been shown to increase the metastatic potential of various types of cancer cells, including prostate cancer cells^[6-10]^. CD44 interactions with various ligands including hyaluronic acid, osteopontin (OPN), matrix metalloproteinases (MMPs), and collagens^[11-13]^ play a crucial role in cancer cell migration and invasion. Specifically, the CD44-osteopontin interaction regulates cell migration to and invasion at distant sites^[12]^. Osteopontin has also been shown to increase both standard and variant CD44 expression in prostate cancer^[14]^. Additionally, the interaction of CD44 with the proteolytic form of MMP-9 is involved in the invasion of PC3 cells^[6]^.

We have previously shown the expression levels of CD44 in different prostate cancer cell lines including LNCaP, DU145, PC3, and PCa2b^[6,15-17]^. The CD44 standard (CD44s) and variant isoforms are cleaved by sequential proteolytic cleavage. This process of sequential cleavage is mediated first by MMPs, generating soluble CD44 fragments or membrane-bound CD44 extracellular truncation (CD44-EXT), followed by intramembrane cleavage by γ-secretase, resulting in the release of the CD44 intracellular domain (CD44-ICD) fragment^[18-20]^. CD44-ICD can translocate to the nucleus, where it regulates the transcription of genes including those encoding MMP-9 and CD44^[21]^. Our most recent studies demonstrated that CD44 can be cleaved by γ-secretase, which results in CD44-ICD formation. DAPT, a γ-Secretase inhibitor may block CD44 cleavage and hence CD44-ICD formation^[22]^. CD44-ICD has been shown to interact with the master regulator of osteoblastogenesis, RUNX2, in the nucleus of breast cancer cells^[21]^. We also previously identified a functional association between RUNX2 and CD44-ICD in PC3 cells in which CD44-ICD localization was increased in the nucleus of PC3 cells overexpressing RUNX2^[22]^.

RUNX2, a transcription factor, plays multiple roles in cancer progression^[23-25]^. RUNX2 regulates the transcription of genes such as *MMP2* and *MMP-9*. Knockdown of RUNX2 decreased the expression of MMP-9 but not MMP2 in PC3 cells^[16,26]^. Furthermore, CD44 regulates the phosphorylation of RUNX2, which is essential for RANKL expression in prostate cancer cells^[15]^. RUNX2 nuclear localization was increased in prostate cancer tissue sections, indicative of a possible predictor of prostate cancer metastasis^[27]^. This study aimed to identify the ability of the CD44-ICD sequence to activate the transcription of a metastatic protein of interest through its interaction with RUNX2, which would provide a mechanism for increasing its different functional potential.

## Methods

### Materials

We obtained antibodies to CD44 [156-3C11], RUNX2 [D1L7F], SOX2 [D6D9], MMP-9 [D6O3H], green fluorescent protein (GFP) [D5.1], and nucleoporin [C39A3] from Cell Signaling Technology, Inc. (Danvers, MA, USA). RUNX2 mouse monoclonal antibody (sc-390351) was purchased from Santa Cruz Biotechnology, Inc. CD44-ICD antibody (KAL-KO601) was purchased from Cosmo Bio. Antibodies against CD44 (ab157107) and GFP (ab1218) were purchased from Abcam. Chemicals and GAPDH antibody (G9545) were purchased from Sigma-Aldrich, Inc. (St. Louis, MO, USA). Horseradish peroxidase-conjugated anti-rabbit and anti-mouse secondary antibodies were obtained from Kirkegaard & Perry Laboratories (Gaithersburg, MD, USA) and Santa Cruz Biotechnology, respectively. Protein assay reagents, molecular weight protein standards, and polyacrylamide gel electrophoresis (PAGE) reagents were purchased from Bio-Rad (Hercules, CA, USA). Polyvinylidene difluoride membranes were obtained from Millipore Corp. (Bedford, MA, USA). Enhanced chemiluminescence reagent was purchased from Pierce (Rockford, IL, USA). Fluorochrome-conjugated secondary antibodies Alexa Fluor 488 (4412) and ProLong Gold Antifade DAPI (8961) were obtained from Cell Signaling Technology, Inc.

### Generation of untagged-CD44-ICD and enhanced green fluorescent protein-CD44-ICD deletion constructs

We utilized a cloning approach to generate untagged and CD44-ICD tagged with GFP and CD44-ICD C-terminal deletion (truncated) constructs. We designed polymerase chain reaction (PCR) primers for the amplification of the human sequence corresponding to CD44-ICD (CD44 Ala^288^ to the stop codon following Val^361^). The primers used are listed below:

Forward Primer: 5ʹ-CCGGAATTCAGGATGGCAGTCAACAGTCGAAGAAGGTGTGG-3

Reverse Primer: 5ʹ-CCGGAATTCCACCCCAATCTTCATGTCCACATTC-3

To generate the CD44-ICD construct, we first PCR-amplified CD44-ICD using the CD44H (CD44-Human; UniProt identifier number P16070-1) sequence as a template and introduced Xho1 and EcoR1 restriction digest sites in the process. The PCR product was subcloned into pcDNA3.1 (-).

To generate CD44-ICD containing enhanced green fluorescent protein (EGFP) at the C-terminal (3’ end), the PCR-amplified untagged CD44-ICD sequence above was PCR-amplified, including the start site and Kozak sequence from the pcDNA3.1 (B) vector and sub-cloned into a pcDNA3-EGFP vector (Addgene). The primer pairs used were:

Forward Primer: 5ʹ-CCCAAGCTTGCAGTCAACAGTCGAAGAAGGTGTGG-3ʹ

Reverse Primer: 5ʹ-CCGGAATTCCACCCCAATCTTCATGTCCACATTC-3ʹ

The amplified PCR product was then subcloned into a pcDNA3-EGFP vector digested with HindIII and EcoR1 enzymes, and we sequentially generated C-terminal deletions (truncations).

### Cloning strategy to generate CD44-ICD untagged and CD44-ICD-EGFP

PCR products were amplified using primers with Xho1 and EcoR1 restriction digest sites and cloned into the pcDNA3.1 (-) vector. Double restriction digestion using HindIII and EcoRI restriction enzymes removed the untagged CD44-ICD from the pcDNA3.1 (-) vector. The insert was then subcloned into an open pcDNA3-EGFP vector to generate an EGFP-CD44-ICD with an EGFP tag at the C-terminus.

### Expression of CD44-ICD constructs in PC3 cells

PC3 cells were grown in 6-well plates overnight in a 37 °C incubator. Once the cells reached ~80% confluency, we transfected them with untagged CD44-ICD and CD44-ICD-EGFP constructs using Lipofectamine 2000 (ThermoFisher Scientific) reagent. The cells were washed and fresh Roswell Park Memorial Institute (RPMI)-1640 medium supplemented with 10% fetal bovine serum (FBS) was added 24 h post-transfection with cDNA. After an additional 24 h, we collected cell lysates, determined protein concentration, and subjected the lysates to sodium dodecyl sulfate (SDS)-PAGE. We performed Western blotting analysis to determine the expression of the CD44-ICD constructs. We continued the stable selection for 3 weeks in 500 μg/mL G418 (product number 30-234-CR; Corning Inc., Corning, NY).

### Cell culture

LNCaP, PC3, and PC3 cells expressing CD44-ICD constructs were cultured in RPMI medium containing 10% FBS as previously described^[6,15]^. The medium was additionally supplemented with 1% PenStrep (penicillin and streptomycin), and the cells were maintained at 37 °C in an incubator with 5% CO_2_.

### RNA extraction and quantitative real-time PCR

We extracted RNA from PC3 cells and PC3 cells expressing CD44-ICD constructs using an RNeasy Midi kit (Qiagen, Valencia, CA, USA) and performed real-time PCR analysis as previously described^[16,17]^. SYBER Green PCR Master Mix (Applied Biosystems) was used along with custom PCR primers (CD44 F: 5ʹ-ACCGACAGCACAGACAGAATC-3ʹ, R: 5ʹ-GTTTGCTCCACCTTCTTGACTC-3ʹ^[17]^; RUNX2 F: 5ʹ-CGGCCCTCCCTGAACTCT-3ʹ, R: 5ʹ-TGCCTGCCTGGGGTCTGTA-3ʹ^[16]^; MMP-9 F: 5ʹ-CTGTCCAGACCAAGGGTACAGCCT-3ʹ, R: 5ʹ-GAGGTATAGTGGGACACATAGTGG-3ʹ^[28]^; OPN F: 5ʹ-CCACAGTAGACACATATGATGG-3ʹ, R: 5ʹ-CAGGGAGTTTCCATGAAGCCAC-3ʹ^[29]^; SOX2 F: 5ʹ-AACCCCAAGATGCACAACTC-3ʹ, R: 5ʹ-CGGGGCCGGTATTTATAATC-3ʹ^[17]^; and GAPDH F: 5ʹ-TGCACCACCAACTGCTTAG-3ʹ, R: 5ʹ-GATGCAGGGATGATGTTC-3ʹ^[16]^.

### Lysate preparation and immunoblotting analysis

The cells were solubilized using lysis buffer containing 62.5 mmol/L Tris-HCl, pH 7.5, 10% glycerol, and 2% SDS^[22]^. The lysates were sonicated for 30 s, centrifuged for 5 min at 14,000 rpm at room temperature, and the supernatants collected. The supernatants were used for protein assay and immunoblotting analyses, as previously described^[17,22]^.

### Preparation of cytoplasmic and nuclear protein fractions

We isolated nuclear and cytoplasmic fractions from the prostate cancer cell lines of interest using a nuclear extraction kit from Abcam (ab112474) according to the manufacturer’s recommendations.

### Immunoprecipitation analysis

Immunoprecipitation (IP) analysis was performed using equal amounts of total or nuclear protein lysates (~50-150 μg) as previously described^[30,31]^.

### Immunostaining analysis

Cell staining and imaging analyses of immunostained cells were performed as previously described^[17]^. Antibodies were diluted in antibody dilution buffer consisting of 1x phosphate-buffered saline (PBS), 1% bovine serum albumin (BSA), and 0.3% Triton X-100. The antibody dilutions were 1:100 (RUNX2), 1:1000 (GFP), and 1:500 (fluorochrome-conjugated FITC, CY2, or CY3 secondary antibodies). The stained cells were imaged using a Nikon W-1 spinning disk confocal microscope. The images were saved and stored in TIF format and processed using Adobe Photoshop (Adobe Systems Inc., Mountain View, CA, USA).

### Immunohistochemistry

We purchased prostatic adenocarcinoma tissue microarray (TMA) sections that contained ten cases of prostate adenocarcinoma and two adjacent normal prostate tissues in duplicate cores per case (US Biomax, Inc., Rockville, MD, USA). The sections were processed as previously described^[16,32]^. Briefly, antigen retrieval was performed in a microwave for 20 min with a buffer containing 10 mmol/L Tris base, pH 9, 1 mmol/L ethylenediaminetetraacetic acid, and 0.05% Tween 20. The sections were incubated in 3% hydrogen peroxide in PBS for 30 min, washed with PBS, and blocked in 2.5% (BSA in PBS for 1 h at room temperature. We incubated sections with primary antibodies that were first diluted in blocking solution overnight at 4 °C. The next day, the slides were washed with PBS and then incubated with secondary biotinylated antibodies (1:500 dilutions) for 1 h, followed by the avidin-biotin complex (ABC) method using an ABC kit (Vector Laboratories, Burlingame, CA, USA) for 30 min. The slides were washed and developed in 3, 3-diaminobenzidine for 2-3 min. The sections were counter-stained with hematoxylin, dehydrated, and then mounted with Permount (Fisher Scientific). The sections were then scanned using an Aperio ScanscopeW CS instrument (Vista, CA, USA). Two investigators semi-quantitatively analyzed the relative distributions of proteins of interest immunostained in the TMA sections.

### Wound closure

Wound closure was performed as previously described^[17]^. Mitomycin C (10 μg/mL) was added to the cell culture medium to prevent cell proliferation during migration in the wound healing assay^[6,17]^. Wound healing/closure was monitored by assessing the migration of cells for 24 h; photographs were taken at 0 and 24 h with a digital SPOT camera attached to an inverted Nikon phase-contrast microscope. The images were stored in TIF format and processed in Adobe Photoshop (Adobe Systems Inc., Mountain View, CA, USA).

### Chromatin immunoprecipitation assay

The chromatin immunoprecipitation (ChIP) assay was performed using kits (catalog numbers 17-295 and 17-371, Millipore Sigma, Burlington, MA, USA) following the manufacturer’s protocol. RUNX2 antibody (sc-390351) with mouse immunoglobulin G as a negative control was used to perform the ChIP assay. The primers used to amplify DNA fragments corresponding to a region on the human MMP-9 promoter^[21]^ were Forward: 5ʹ-‘AGGTACCACAGTTCCCACAAGCTCTGC-3’ʹ, Reverse: 5ʹ-‘TTAAGCTTGGAGCACCAGGACCAGGG-3’ʹ^[21]^.

### Statistical analysis

Values are presented as mean ± standard error of the mean (SEM). *P* < 0.05 was considered statistically significant. Two-tailed Student’s *t*-tests or one-way analysis of variance (ANOVA) was used to determine significance. Data were analyzed with GraphPad Prism Software (La Jolla, CA, USA).

## Results

### Prostate cancer PC3 cells highly express CD44, CD44-ICD, and RUNX2 proteins, which colocalize in the nucleus

**Figure 1:**
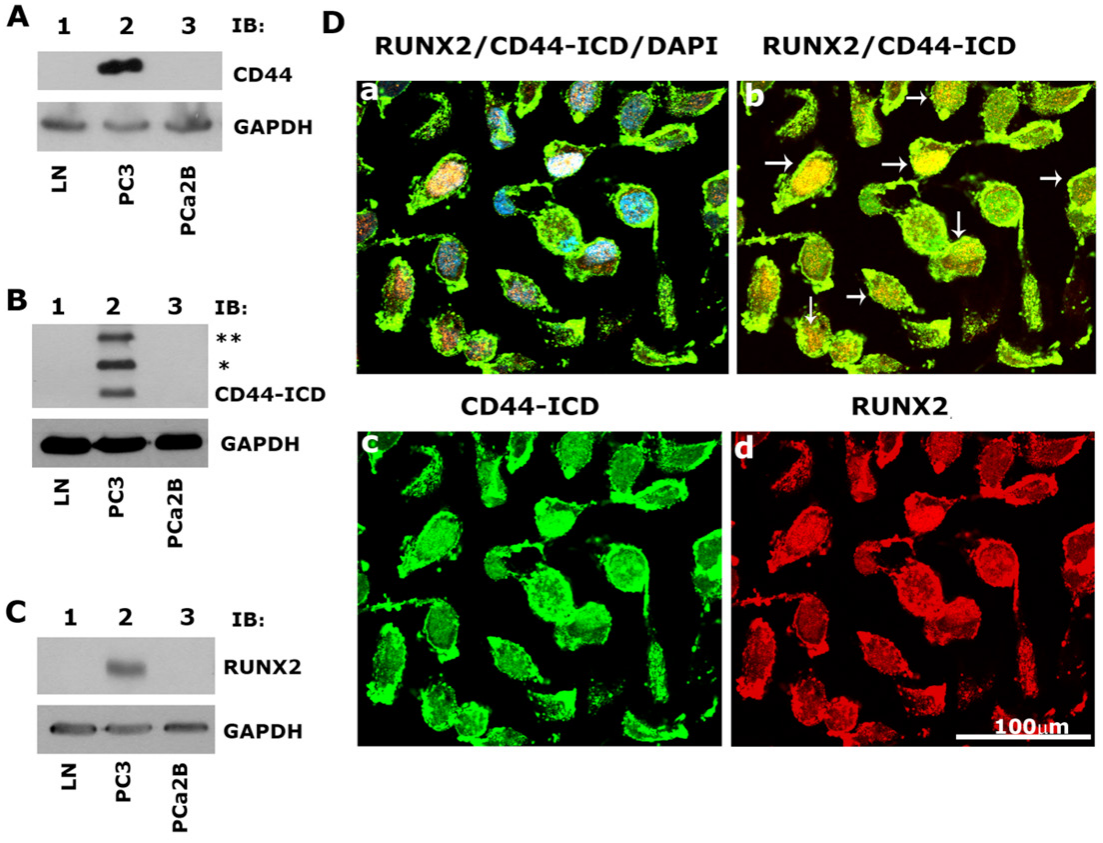
Immunoblotting and confocal microscopy analysis of the expression and distribution of CD44, CD44-ICD and RUNX2 in PCa cell lines. A-C: an equal amount of protein lysates (40 µg) made from LNCaP (lane 1), PC3 (lane 2) and PCa2b (lane 3) cells were immunoblotted with CD44 (A), CD44-ICD (B), and RUNX2 (C) antibodies to detect total cellular levels of the respective proteins. (*) and (**) represent the ~ 20-kDa and ~25-kDa fragments of CD44 extracellular truncation fragment (CD44-EXT). CD44-ICD is ~16.5-kDa fragment of CD44. Immunoblotting with a GAPDH antibody was used as a loading control; D: immunostaining analysis of the distribution of RUNX2 (red), CD44-ICD (green), and DAPI (blue). Arrows point to the regions of colocalization (yellow) in RUNX2/CD44-ICD panel. Scale bar: 100 µm. The results represent one of the three separate experiments performed with the same results. CD44: Cluster of differentiation 44; ICD: intracellular domain

As shown previously^[22]^, immunoblotting analyses revealed the expression of CD44, CD44-ICD, and RUNX2 [Figure 1A-C; Lane 2] in PC3 cells as compared to LNCaP [Figure 1A-C, lane 1] and PCa2b [Figure 1A-C, lane 3] cells [Table 1]. Immunostaining analysis followed by confocal microscopy showed colocalization of CD44-ICD and RUNX2 in the nucleus of PC3 cells. Colocalization appears as yellow areas in the nuclei of PC3 cells in Figure 1D (panel b, arrows). DAPI was used to counterstain the nucleus with negligible to no cytoplasmic background staining. Overlay staining demonstrated the colocalization of DAPI (blue) with CD44-ICD and RUNX2 in the nucleus, with colocalization appearing as purple areas in the nuclei of a few cells [Figure 1D, panel a]. These results confirm our previous observations^[22]^ that CD44 cleavage results in nuclear translocation and colocalization with RUNX2 (red, panel d) in areas with intense CD44-ICD staining (green, panel c).

**Table 1:** Cell lines: list of prostate cancer cell lines, derivatives, and androgen receptor status

Cell line	Derivative	Androgen receptor status
PC3	Caucasian bone metastasis	Negative/insensitive
LNCaP	Caucasian lymph node metastasis	Positive/sensitive
MDA PCa2b	African American bone metastasis	Positive/sensitive
PC3/RUNX2	PC3 cells stably expressing RUNX2 cDNA	
PC3/CD44-ICD constructs	PC3 cells stably expressing C-terminal deletion constructs of CD44-ICD	

CD44: Cluster of differentiation 44; ICD: intracellular domain

### High expression of CD44-standard (CD44s) and CD44-ICD in prostatic adenocarcinoma tissue microarray sections

**Figure 2:**
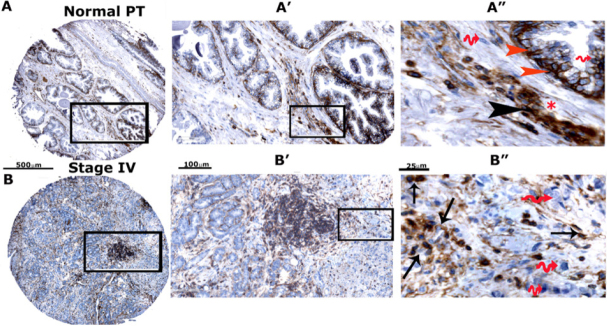
Immunohistochemical analysis of TMA in adjacent normal prostate tissue and adenocarcinoma (stage IV). Immunohistochemical staining was performed with an antibody to CD44-ICD in prostate cancer tissue array with adjacent normal prostate tissue. Sections were then scanned using an Aperio Scanscope^®^ CS instrument (Aperio Scanscope CS system, Vista, CA, USA). A, B: represent normal prostatic and adenocarcinoma (stage IV) tissue sections, respectively. These sections are magnified in A’, A’’, B’ and B’’. Staining was repeated two times. Scale bar represents 500 µm (A and B), 100 µm (A’ and B’), and 25 µm (A” and B”); C, D: the protein expression pattern is expressed as percent cells stained per core for CD44-ICD and CD44s proteins and presented as a graph. Data are given as a scatterplot for the indicated number of cores analyzed in Table 2. The number of cores that were analyzed by two investigators are provided in the parentheses of the first column denoted as “Grade” in Table 2. CD44: Cluster of differentiation 44; ICD: intracellular domain

To further validate our immunoblotting findings, we compared the expression levels of CD44s and CD44-ICD in prostate cancer tissue microarrays [Figure 2 and Supplementary Figures 1 and 2]. Using microarray sections (two PR242a and one PR243 from Biomax) containing six cases of prostate adenocarcinoma and six adjacent normal prostate tissues with duplicate cores for each case, we performed an immunohistochemical analysis with antibodies to CD44-ICD [Supplementary Figure 1A and B]. The relative distributions of CD44-ICD and CD44s in stained TMA sections were semi-quantitatively analyzed by two investigators [Table 2]. CD44-ICD was observed predominantly in the nuclei of basal cells [red arrowheads; Figure 2A] and stromal cells [black arrowheads; Figure 2A] of normal prostate cells. Very little staining was observed in the epithelial cells of the lumen. Although the lumen is filled with adenocarcinoma cells, few cells in the lumen displayed the distribution of CD44-ICD [Figure 2B]. The nuclear distribution of CD44-ICD was magnified in cancer cells disseminating from the lumen [arrows, Figure 2B]. The cytoplasmic distribution of CD44-ICD was very sparse. The wavy red arrows in Figure 2 [panels A” and B”] point to the nuclei of basal, stromal, and carcinoma cells with no CD44-ICD staining. In contrast, although CD44s was distributed in both normal and prostatic adenocarcinoma cells, staining was intense in sections containing adenocarcinoma (grade I to III) because the lumens were filled with adenocarcinoma cells [Supplementary Figure 2A-C]. The expression levels of CD44-ICD and CD44s in normal prostatic and adenocarcinoma tissues are summarized in Table 2. The number of cores analyzed for CD44-ICD and CD44s is indicated in the scatter plot of Figure 2C and D. The enrichment of CD44-ICD in the nuclei of cancerous cells may assist in tumor progression via the regulation of transcription of metastasis-related genes (e.g., *OPN*, *RANKL*, and *MMP-9*).

**Table 2:** Expression of CD44-ICD and CD44s in prostatic carcinoma and cancer adjacent to normal prostate tissue sections

Grade	Cells	CD44-ICD	CD44s
Normal prostatic epithelial cells and PCa adjacent to these cells (*n* = 12)	Cancer cells appear normal cells (NC) Normal stromal cells	NC* = 6.8% ± 3.2% PCa = 4.13% ± 1.5% Stromal cells < 5%	NC* = 22.7% ± 11.04% PCa = 7.13% ± 3.23% Stromal cells < 5%
Adenocarcinoma (Type: Malignant) Grade 1 (*n* = 8)	Cells appear slightly different than normal; moderately differentiated with normal stromal cells	PCa = 5.00% ± 1.4% Stromal cells < 4%	PCa = 41.5% ± 19.22% Stromal cells ~8%
Adenocarcinoma with necrosis (Type: Malignant) Grade 2 (*n* = 16)	Cells appear abnormal; poorly differentiated; stroma is less	PCa = 10.13% ± 2.4%* Stromal cells ~8%	PCa = 58.00% ± 12%** Stromal cells ~5%-7%

Prostatic carcinoma and normal tissue microarray containing 12 cases/24 cores was used. Immunohistochemistry was performed with an antibody to CD44s and CD44-ICD. Staining was done in duplicate with two different microarrays (PR243 and 243a; Biomax). The number of cores that were analyzed by two investigators is provided in the parentheses in the 1st column denoted as “Grade”. Percent staining in each core is presented in a scatter plot (Figure 2C). *P < 0.01 and **P < 0.001 staining intensity *vs*. normal cells. CD44: Cluster of differentiation 44; CD44s: CD44 standard; ICD: intracellular domain

### Overexpression of the CD44-intracellular domain increases expression of metastasis-related genes and cell migration in PC3 cells

**Figure 3:**
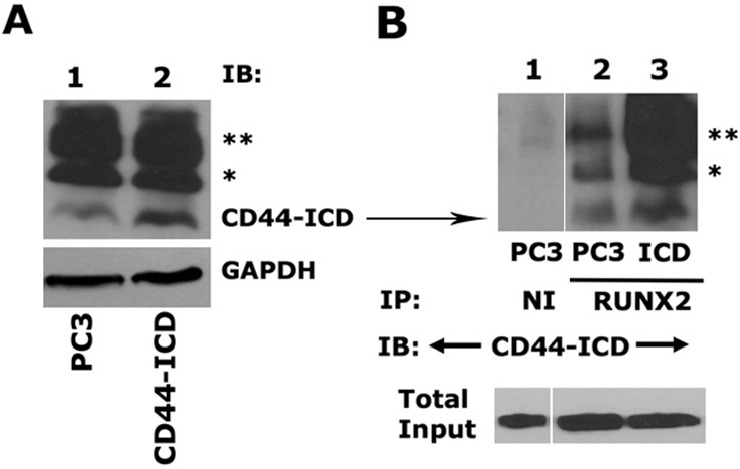
Analysis of CD44-ICD overexpression and its interaction with RUNX2. A: an equal amount of protein lysates (40 μg) prepared from PC3 cells transfected with CD44-ICD or control PC3 cells were used for immunoblotting analysis with a CD44-ICD antibody. Immunoblotting with a GAPDH antibody was used as a loading control; B: equal amounts of PC3 lysates (200 µg) were immunoprecipitated with a RUNX2 antibody (lane 2-3) or a species-specific non-immune serum (NI, lane 1). Immunoprecipitates were subjected to immunoblotting with an antibody to CD44-ICD. (*) and (**) represent the ~20-kDa and ~25-kDa fragments of CD44 extracellular truncation fragment (CD44-EXT). CD44-ICD is ~16.5-kDa fragment of CD44. An equal amount of lysate (Input) used for immunoprecipitation was assessed by direct immunoblotting of lysates with an antibody to nucleoporin. CD44: Cluster of differentiation 44; ICD: intracellular domain

We next determined the effect of CD44-ICD overexpression on the expression of metastasis-related genes (SOX2, MMP9, and OPN) and cell migration in PC3 cells after stable transfection. As described in the Methods section, an immunoblotting analysis was performed with an anti-CD44-ICD antibody to determine the expression levels in PC3 and PC3 cells transfected with the CD44-ICD construct [Figure 3A]. CD44-ICD overexpression was observed at fragment molecular weights of ~16.5 kDa, ~20 kDa, and ~25 kDa in PC3 cells transfected with CD44-ICD [Figure 3A, lane 2] as compared to those in control PC3 cells (lane 1). This overexpression corresponded to increased co-precipitation of CD44-ICD fragments in immunoprecipitate made with a RUNX2 antibody [Figure 3B, lane 3]. CD44-ICD expression was not observed in immunoprecipitates occurring with a species-specific non-immune serum (NI) [Figure 3B, lane 1]. Co-precipitation of all CD44-ICD fragments (~16.5-kDa, ~20-kDa, and ~25-kDa fragments) with RUNX2 immunoprecipitation suggests their binding specificity for the RUNX2 protein.

**Figure 4:**
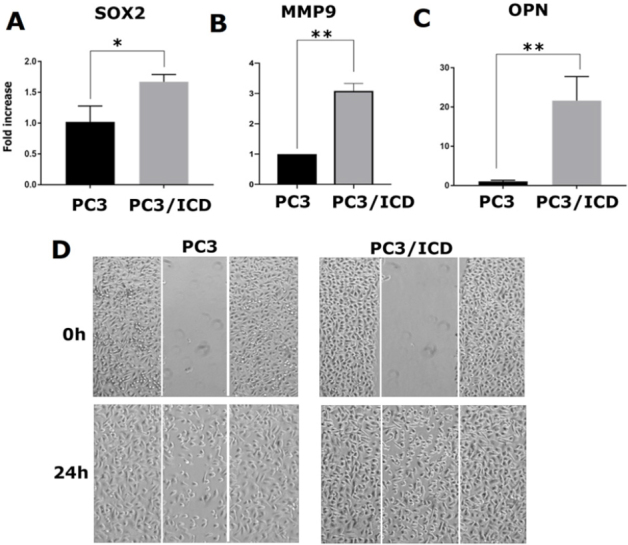
Analysis of the effect of CD44-ICD overexpression on the expression of metastasis-related genes and migration in PC3 cells. Real-time PCR analysis of SOX2 (A), MMP-9 (B) and OPN (C) expression in PC3 and PC3-CD44-ICD cells. GAPDH was used as a loading control for real-time PCR analysis. PC3 (left panel) and PC3 cells transfected with CD44-ICD (right panel) were subjected to wound-closure assay. Phase contrast micrographs show migration at 0 and 24 h (D). Scale bar: 200 µm. The results shown are representative of three independent experiments. *P < 0.05 or **P < 0.01. CD44: Cluster of differentiation 44; ICD: intracellular domain

RUNX2 is abnormally expressed in prostate cancer cells (PC3) and, to a lesser extent, in LNCaP cells^[16,22,26]^. In a metastasis model, high RUNX2 levels were shown to increase the expression of several metastasis-related genes (e.g., MMP9, MMP13, vascular endothelial growth factor, and OPN) and secreted bone resorption factors (e.g., parathyroid hormone-related protein and interleukin 8), which promote osteolytic disease^[26]^. Here, we evaluated whether CD44-ICD overexpression increased the expression levels of any metastasis-related genes via its interaction with RUNX2 [Figure 3B]. Our initial characterization indeed demonstrated increased expression of metastasis-related genes such as *SOX2* [Figure 4A], *MMP-9* [Figure 4B], and *OPN* [Figure 4C] at the mRNA level in cells overexpressing CD44-ICD.

To analyze the functional role of CD44-ICD overexpression, we performed wound-healing assays in PC3 and PC3/CD44-ICD-overexpressing cells. The cells were pretreated with mitomycin C to ensure that changes in cell migration were independent of cellular proliferation [Figure 4D]. The increased wound-closure capacity in cells expressing CD44-ICD may be due to the expression of the above genes. Our observations suggest that interactions between CD44-ICD and RUNX2 may be critical for the expression of metastasis-related genes. Thus, CD44-ICD may function as a co-factor in RUNX2-mediated transcriptional processes.

### Overexpression of CD44-ICD-EGFP deletion (truncated) constructs in PC3 cells alters PC3 cell morphology

**Figure 5:**
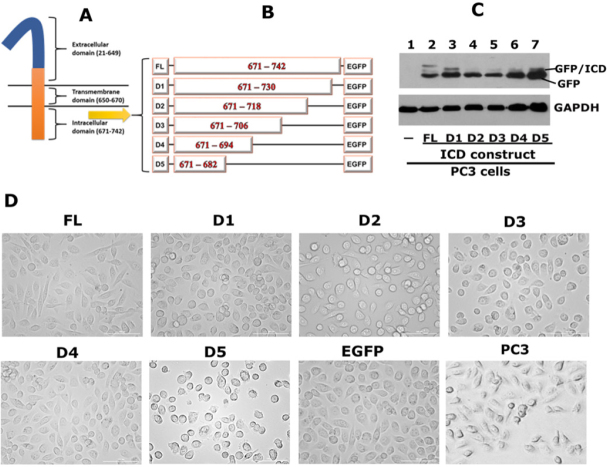
Generation of CD44-ICD-EGFP plasmid constructs and transfecting into PC3 cells. A: a schematic diagram of CD44 domains is provided; B: a schematic diagram of constructs generated is shown. Full-length CD44-ICD (FL) and deletion constructs of CD44-ICD (D1-D5) were generated in EGFP vector. Numbers in the figure indicate the amino acid (aa) sequence for each construct; C: immunoblotting analysis using anti-GFP antibody is shown. Immunoblotting with a GAPDH antibody was used as a loading control. GFP/ICD fusion proteins of different sizes and GFP (~27-29 kDa) are indicated in Figure C; D: Phase contrast micrograph shows the morphology of PC3 cells transfected with indicated CD44-ICD-constructs and EGFP vector as well as untransfected PC3 cells. Magnification 100X. CD44: Cluster of differentiation 44; ICD: intracellular domain

The extracellular, transmembrane and intracellular domains of CD44 are indicated in the diagrammatic sketch shown in Figure 5A. We demonstrated the interaction of CD44-ICD with RUNX2 in the nucleus of PC3 cells [Figure 1]. We then mapped the CD44-ICD sequences, which demonstrated specific interactions with RUNX2. Deletion constructs were generated by sequential ~12 amino acid (~36 nucleotide) deletions of the full length (FL) ICD fragment (671-742 aa) in the EGFP vector, as shown in the schematic diagram in Figure 5B. The deletion constructs are denoted as D1–D5 and full-length as FL-ICD.

We generated stable PC3 cell lines expressing the constructs of interest [Figure 5A]. Immunoblotting analysis demonstrated the successful expression of GFP-fusion proteins with the expected molecular weights. A protein band with a molecular weight of approximately 45-49 kDa represents the GFP-fused FL-ICD protein [Figure 5C, lane 2]. A size-wise decrease in the molecular weight of the fusion proteins was observed in cells expressing the D1-D5 constructs [Figure 5C, lane 3-7]. We also observed the expression of free GFP protein with a molecular weight corresponding to 29-30 kDa [Figure 5C, lanes 2-7]. Untransfected PC3 cells negative for GFP expression were used as controls [Figure 5C, lane 1].

Phase-contrast microscopy revealed the morphology of untransfected and transfected PC3 cells. As shown previously^[17]^, untransfected PC3 cells are slightly spindle-shaped compared to transfected cells. However, transfected PC3 cells with ICD constructs did not show any significant differences compared to vector (EGFP)-transfected cells [Figure 5D]. Untransfected or transfected cells were not deficient in adhesion and spread well on the culture dish.

### Analyses of the specificity of the interaction of RUNX2 with ICD sequences

**Figure 6:**
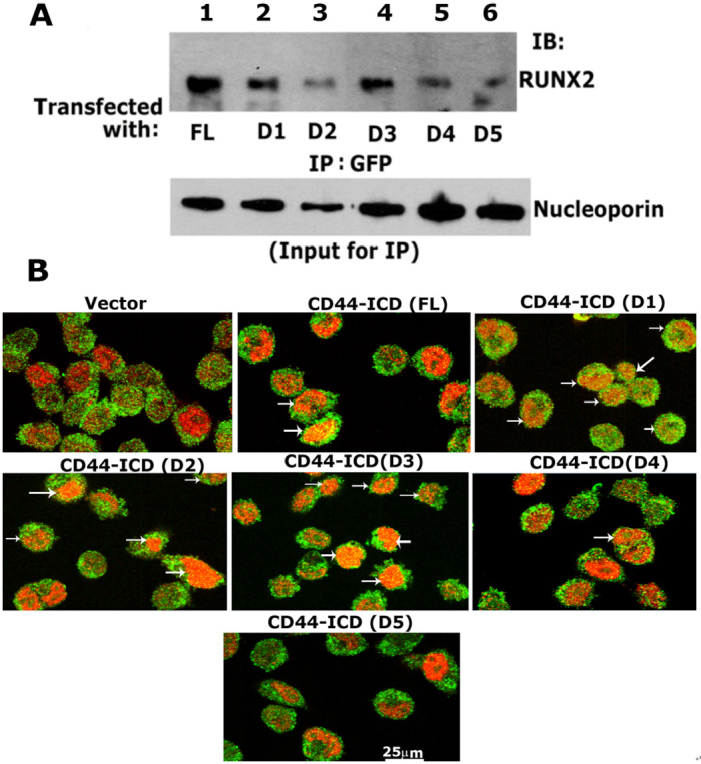
Analysis of the specificity of interaction of RUNX2/CD44-ICD deletion constructs. A: immunoblotting analysis with a RUNX2 antibody (top panel): Nuclear lysates prepared from PC3 cells transfected with indicated constructs were immunoprecipitated with a GFP antibody (lane 1-6) and immunoblotted (IB) with an antibody to RUNX2 (lanes 1-6). An equal amount of nuclear lysate used for immunoprecipitation (IP) was assessed by direct immunoblotting of lysates with an antibody to nucleoporin (Input for IP). A decrease in loading was observed in D2 samples, which corresponded to the possible decrease in the coprecipitation of RUNX2; B: confocal microscopy analyses of cells transfected with CD44-ICD deletion construct and stained with an antibody to GFP (green) and RUNX2 (red). Colocalization is seen in yellow (indicated by arrows in FL, D1, D2, D3, and D4). Scale bar: 25 µm. CD44: Cluster of differentiation 44; ICD: intracellular domain

Having generated stable PC3 cell lines with the constructs of CD44- ICD, we then determined the sequences that showed specific interactions with RUNX2 by co-immunoprecipitation followed by conventional immunoblotting analyses [Figure 6]. The nuclear fractions of cells co-expressing GFP-ICD (FL and D1-D5) were used for immunoprecipitation with a GFP antibody and immunoblotted with a RUNX2 antibody. Co-precipitation of RUNX2 was observed in the nuclear lysates from cells transfected with FL [Figure 6A, lane 1], D1 (lane 2), D2 (lane 3), and D3 (lane 4). RUNX2 co-precipitation was very low in cells transfected with D4 and D5 constructs (lanes 5 and 6). Equal loading (total input) was assessed by direct immunoblotting of lysates with a nucleoporin antibody. The reduced co-precipitation of RUNX2 in the D3 construct was due to the amount of lysate used and was considerably lower for immunoprecipitation. This can be seen in the direct immunoblotting analysis of total lysates with a nucleoporin antibody.

To further corroborate these findings, we performed immunostaining analysis with an antibody against GFP and RUNX2 [Figure 6B and [Supplementary Figure 3]. A rectangular field [Supplementary Figure 3] corresponded to the areas of magnification in Figure 6B. GFP expression was observed in the membrane, nucleus, and cytoplasm of cells expressing the vector and ICD constructs [Figure 6B and Supplementary Figure 3]. However, colocalization (yellow) with RUNX2 (red) was observed in cells expressing CD44-ICD (FL) and deletion constructs of ICD (D1-D3). The punctate yellow color in the nucleus represented colocalization (arrows) of RUNX2 (red) and indicated ICD protein (green). Colocalization was minimal or not observed in cells expressing the D4 and D5 constructs.

### Sequence-specific interactions of CD44-ICD/RUNX2 at the promoter region of the *MMP-9* gene

**Figure 7:**
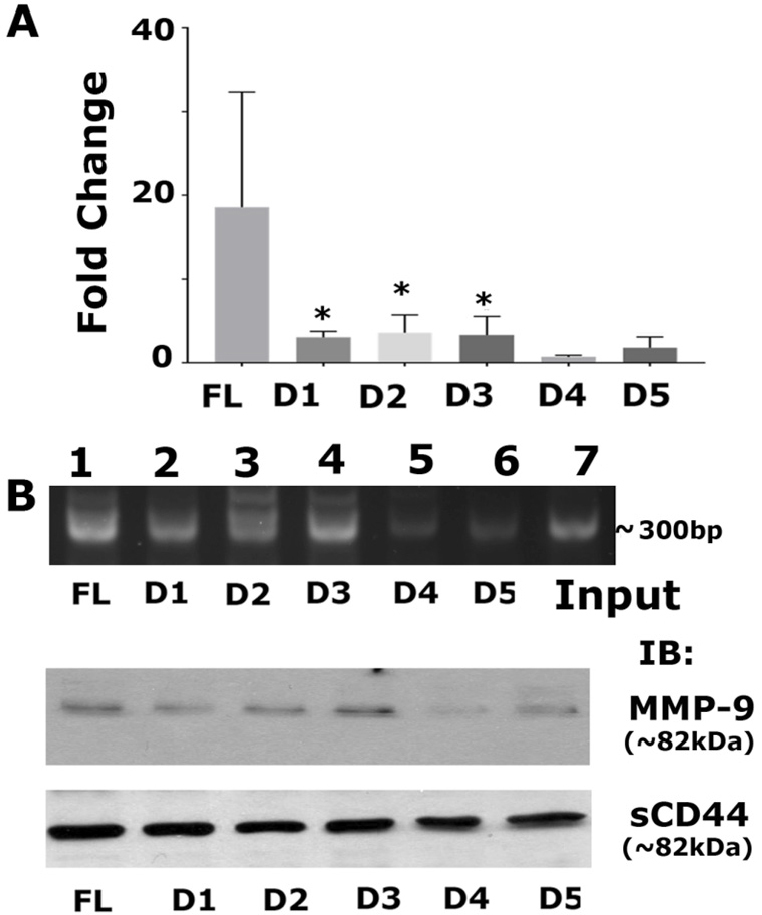
Analysis of mRNA expression of MMP-9 and the effect of sequence-specific interaction of CD44-ICD/RUNX2 on the promoter region of the MMP-9 gene. A: real-time PCR analysis of MMP-9 expression was done in PC3 cells expressing indicated CD44-ICD deletion constructs. GAPDH was used as a loading control for real-time PCR analysis; B: ChIP assay (top): ChIP assay was performed in cells expressing indicated constructs for MMP-9 promoter. ChIP assay showed an increase in signal in PC3 cells expressing FL, D1, D2, and D3. Signal is considerably reduced in D4 and D5. Immunoblotting analysis with an antibody to MMP9 is shown (middle panel in B). The expression levels of MMP-9 protein corresponded to the observations shown in ChIP assay. Immunoblotting with a sCD44 antibody was used as loading control (B; bottom panel). *P < 0.05 *vs*. D4 and D5. CD44: Cluster of differentiation 44; ICD: intracellular domain

We aimed to determine if the sequence-specific deletion construct had a greater affinity for interacting with RUNX2 on the promoter region of any of the metastasis-related genes (*MMP-9*). Recent reports have identified the interaction of RUNX2 with CD44-ICD on the promoter of the *MMP-9* gene in breast cancer^[21]^. Therefore, we measured MMP-9 protein and mRNA expression levels and observed higher mRNA expression of MMP-9 in CD44-ICD deletion constructs [Figure 7A]. Furthermore, RUNX2 binding to the MMP-9 promoter was higher in cells expressing FL-ICD and D1–D3 constructs [Figure 7B]. MMP-9 protein levels corroborated the observation [Figure 7B, middle panel] that CD44-ICD sequences 694-706 have more binding specificity towards RUNX2. Truncation/deletion of these sequences in D4 and D5 did not regulate MMP-9 expression. We believe that this sequence-specific interaction is required for the promoter activity of MMP-9. We used deletion constructs to map the specific binding sequences of ICD with RUNX2 as for initial and primary characterization. Further delineation is required to determine whether this interaction is cell-specific and could be a novel therapeutic target for metastatic cancer cells.

## Discussion

The role of CD44 signaling in metastatic processes has been studied extensively. However, little is known about the mechanisms involved or related to cancer progression when CD44 undergoes proteolytic cleavage to CD44-ICD. Several studies have shown that CD44 proteolytic cleavage is linked to cancer progression and metastasis^[21,33]^, demonstrating that the release of CD44 is associated with presenilin-dependent γ-secretase and membrane-associated metalloprotease activity. These proteolytic activities result in CD44-ICD formation, which may induce the transcriptional activation of the genes of interest. Genes regulated by CD44-ICD as a transcriptional or co-transcriptional factor could regulate tumor progression. CD44-ICD has been shown to regulate the expression of numerous genes via its interaction with RUNX2 in breast cancer cells. One such consequence of CD44-ICD cleavage is the generation of several fragments. Soluble CD44 (sCD44) can be generated from the proteolytic cleavage of CD44. The release of soluble ECD of CD44 into the serum may be an indicator of tumor progression and metastasis in colon cancer. sCD44 has also been shown to be a valuable indicator of tumor growth in colorectal and gastric cancers^[18-21,34-37]^.

Previous studies and the present one from our laboratory have shown that CD44 is an important regulator of tumor progression in prostate cancer^[6,15-17,22]^. Previously, we demonstrated the cleavage of CD44 and the role of the cleavage product (CD44-ICD) as a transcriptional/co-transcriptional factor with RUNX2 in PC3 cells^[22]^. Ultimately, it is essential to identify (1) if there are interactions between RUNX2 and CD44-ICD that could affect the promoter activity of the genes of interest (e.g., MMP-9); and (2) the specificity of the CD44-ICD sequence involved in this interaction. Therefore, our goal in the present study was to generate CD44-ICD truncation/deletion constructs using conventional recombinant DNA techniques and identify the CD44-ICD constructs that interacted with RUNX2 and transcription of genes of interest. We used appropriate transfection methods in PC3 cells to test the effect of the expression of full-length CD44-ICD and deletion constructs of CD44-ICD.

We first reconfirmed the expression levels of CD44, CD44-ICD, and RUNX2 in three significant prostate cancer cell lines (LNCaP, PC3, and PCa2b). CD44 and RUNX2 expression was observed in PC3 cells and the interaction of CD44-ICD with RUNX2 was observed in the nuclei of PC3 cells, which are androgen receptor-negative. Second, we determined the expression levels of CD44-ICD in tissue microarray sections containing normal prostatic tissues and stage 1-4 adenocarcinoma tissue. RUNX2 functions as a key transcription factor in osteoblastogenesis and is highly expressed in cancers as well as human tissue microarrays in both adenocarcinoma and metastasis^[26]^. We have previously shown that RUNX2, CD44s, and MMP9 are highly expressed in tumor tissue. Stage 1-4 prostatic adenocarcinoma showed higher expression levels of these metastasis-related proteins, although the expression of these proteins was also observed in normal prostatic tissue to a lesser extent. Increased expression was partly due to the lumen of the prostatic tumor tissue filling with adenocarcinoma cells^[16,38]^.

As shown previously^[15,25,39,40]^, CD44s staining observed in adenocarcinoma cells present in the lumen was distributed in the membrane, nuclei, and (weakly) in the cytoplasm of these cells. CD44-ICD staining was perinuclear and predominantly in the nuclear regions of adenocarcinoma cells as well as in the nuclei of some basal and stromal cells. Furthermore, staining was observed only in a few cancerous cells exiting or disseminated from the lumen. These cancerous cells also showed epithelial-mesenchymal transition-like phenotypes (i.e., about to leave the lumen). The predominant localization of CD44 in the nuclei of luminal normal epithelial prostatic cells and adenocarcinoma cells suggests that it is the cleaved product of CD44, i.e., “CD44-ICD”. Localization of CD44-ICD and RUNX2^[16]^ in the nuclei of adenocarcinoma further highlights the potential role of these proteins in transcriptional regulation and tumor progression. Thus, CD44-ICD could be a useful biomarker of cancer progression.

According to our goal, we first cloned FL CD44-ICD into a pcDNA vector lacking any tag. This construct was later used to generate EGFP-tagged CD44-ICD deletion and FL constructs. We first determined the functional significance of FL-ICD overexpression in wound healing and qPCR analyses. CD44-ICD overexpression increased not only metastasis-related gene (*SOX2*, *MMP-9*, and *OPN*) expression but also wound healing capabilities. These results further highlighted the overall impact of CD44-ICD in mediating tumorigenesis.

These observations prompted us to generate deletion constructs using the FL/CD44-ICD generated in the pcDNA vector described above. We had to overcome some technical challenges in our cloning strategy to express proteins with the expected molecular weight. We cloned and expressed the FL and elements of CD44-ICD in an expression vector containing EGFP and transfected into PC3 cells. Lysates from these transfectants were immunoblotted with an antibody against GFP. PC3 cells stably transfected with the deletion constructs of CD44-ICD and FL/CD44-ICD showed expression of CD44-ICD as GFP-fusion proteins at the expected molecular weight. Surprisingly, these fusion proteins retained their specificity for RUNX2 binding.

Furthermore, we showed in the present study a considerable CD44-ICD/RUNX2 interaction in cells expressing FL-ICD and D-D3 ICD constructs. A similar trend was observed in the mRNA expression levels of MMP-9 by real-time PCR analysis. ChIP assay and immunoblotting analyses corroborated this observation. Although further characterization is necessary, we believe that CD44-ICD sequences between amino acid positions 694 and 706 are a good therapeutic target to reduce the expression of metastasis-related events.

**Figure 8:**
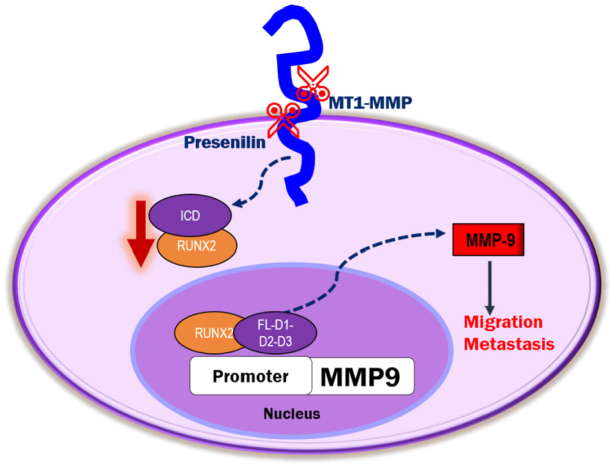
Schematic diagram illustrating the proposed mechanism of CD44-ICD-DelConstruct-RUNX2 interaction in PC3 cells. CD44 is sequentially cleaved to generate CD44-intracellular domain (ICD) fragment. CD44-ICD-FL, D1, D2 and D3 fragments interact with RUNX2 in the nucleus of PC3 cells to activate transcription of MMP-9 to promote tumor progression via migration and metastasis. CD44: Cluster of differentiation 44

To our knowledge, our findings are the first to show the sequence-specific interaction of RUNX2 with ICD on the promoter of the *MMP-9* gene. We propose that the release of CD44-ICD into the cytoplasm and subsequent translocation to the nucleus may regulate the transcription of essential genes involved in metastasis. Additional studies are needed to determine the role of ICD as a co-transcriptional factor in the expression of metastasis-related genes of interest [Figure 8]. Further investigation of the interaction of amino acid sequences 694 to 706 in the CD44-ICD-D3 construct with RUNX2 as a co-factor for the expression of metastasis-related genes is needed. Much remains to be elucidated regarding CD44-ICD signaling, interactions, and tumorigenesis.

## Declarations

### Availability of data and materials

All data generated and analyzed during this study are included in the article. All data are available from the corresponding author upon request.

### Copyright

© The Author(s) 2022.

## References

[1] Siegel RL, Miller KD, Jemal A (2019). Cancer statistics, 2019.. CA Cancer J Clin.

[2] Brawley OW (2012). Prostate cancer epidemiology in the United States.. World J Urol.

[3] Adesunloye BA, Karzai FH, Dahut WL, Marone G, Granata F (2013). Angiogenesis inhibitors in the treatment of prostate cancer.. Angiogenesis, Lymphangiogenesis and Clinical Implications..

[4] Varenhorst E, Klaff R, Berglund A, Hedlund PO, Sandblom G, Scandinavian Prostate Cancer Group (SPCG) Trial No. 5 (2016). Predictors of early androgen deprivation treatment failure in prostate cancer with bone metastases.. Cancer Med.

[5] Frieling JS, Basanta D, Lynch CC (2015). Current and emerging therapies for bone metastatic castration-resistant prostate cancer.. Cancer Control.

[6] Desai B, Rogers MJ, Chellaiah MA (2007). Mechanisms of osteopontin and CD44 as metastatic principles in prostate cancer cells.. Mol Cancer.

[7] Cooper CR, Chay CH, Pienta KJ (2002). The role of alpha(v)beta(3) in prostate cancer progression.. Neoplasia.

[8] Weber GF, Ashkar S (2000). Molecular mechanisms of tumor dissemination in primary and metastatic brain cancers.. Brain Res Bull.

[9] Naor D, Vogt Sionov R, Zahalka M, Rochman M, Holzmann B, Holzmann B, Wagner H (1998). Organ-specific requirements for cell adhesion molecules during lymphoma cell dissemination.. Leukocyte integrins in the immune system and malignant disease..

[10] Sy MS, Guo YJ, Stamenkovic I (1991). Distinct effects of two CD44 isoforms on tumor growth in vivo.. J Exp Med.

[11] Goodison S, Urquidi V, Tarin D (1999). CD44 cell adhesion molecules.. Mol Pathol.

[12] Weber GF, Ashkar S, Glimcher MJ, Cantor H (1996). Receptor-ligand interaction between CD44 and osteopontin (Eta-1).. Science.

[13] Desai B, Ma T, Chellaiah MA (2008). Invadopodia and matrix degradation, a new property of prostate cancer cells during migration and invasion.. J Biol Chem.

[14] Chellaiah MA, Kizer N, Biswas R, Alvarez U, Strauss-Schoenberger J (2003). Osteopontin deficiency produces osteoclast dysfunction due to reduced CD44 surface expression.. Mol Biol Cell.

[15] Desai B, Ma T, Zhu J, Chellaiah MA (2009). Characterization of the expression of variant and standard CD44 in prostate cancer cells: identification of the possible molecular mechanism of CD44/MMP9 complex formation on the cell surface.. J Cell Biochem.

[16] Gupta A, Cao W, Chellaiah MA (2012). Integrin αvβ3 and CD44 pathways in metastatic prostate cancer cells support osteoclastogenesis via a Runx2/Smad 5/receptor activator of NF-κB ligand signaling axis.. Mol Cancer.

[17] Srinivasan D, Senbanjo L, Majumdar S, Franklin RB, Chellaiah MA (2018). Androgen receptor expression reduces stemness characteristics of prostate cancer cells (PC3) by repression of CD44 and SOX2.. J Cell Biochem.

[18] Murakami D, Okamoto I, Nagano O, Kawano Y, Tomita T (2003). Presenilin-dependent gamma-secretase activity mediates the intramembranous cleavage of CD44.. Oncogene.

[19] Okamoto I, Kawano Y, Murakami D, Sasayama T, Araki N (2001). Proteolytic release of CD44 intracellular domain and its role in the CD44 signaling pathway.. J Cell Biol.

[20] Okamoto I, Kawano Y, Tsuiki H, Sasaki J, Nakao M (1999). CD44 cleavage induced by a membrane-associated metalloprotease plays a critical role in tumor cell migration.. Oncogene.

[21] Miletti-González KE, Murphy K, Kumaran MN, Ravindranath AK, Wernyj RP (2012). Identification of function for CD44 intracytoplasmic domain (CD44-ICD): modulation of matrix metalloproteinase 9 (MMP-9) transcription via novel promoter response element.. J Biol Chem.

[22] Senbanjo LT, AlJohani H, Majumdar S, Chellaiah MA (2019). Characterization of CD44 intracellular domain interaction with RUNX2 in PC3 human prostate cancer cells.. Cell Commun Signal.

[23] van der Deen M, Akech J, Wang T, FitzGerald TJ, Altieri DC (2010). The cancer-related Runx2 protein enhances cell growth and responses to androgen and TGFbeta in prostate cancer cells.. J Cell Biochem.

[24] Zhang X, Wu H, Dobson JR, Browne G, Hong D (2015). Expression of the IL-11 Gene in Metastatic Cells Is Supported by Runx2-Smad and Runx2-cJun Complexes Induced by TGFβ1.. J Cell Biochem.

[25] Pratap J, Lian JB, Stein GS (2011). Metastatic bone disease: role of transcription factors and future targets.. Bone.

[26] Akech J, Wixted JJ, Bedard K, van der Deen M, Hussain S (2010). Runx2 association with progression of prostate cancer in patients: mechanisms mediating bone osteolysis and osteoblastic metastatic lesions.. Oncogene.

[27] Chua CW, Chiu YT, Yuen HF, Chan KW, Man K (2009). Suppression of androgen-independent prostate cancer cell aggressiveness by FTY720: validating Runx2 as a potential antimetastatic drug screening platform.. Clin Cancer Res.

[28] Park JS, Park MK, Oh HJ, Woo YJ, Lim MA (2012). Grape-seed proanthocyanidin extract as suppressors of bone destruction in inflammatory autoimmune arthritis.. PLoS One.

[29] Neumann C, Garreis F, Paulsen F, Hammer CM, Birke MT (2014). Osteopontin is induced by TGF-β2 and regulates metabolic cell activity in cultured human optic nerve head astrocytes.. PLoS One.

[30] Chellaiah M, Hruska K (1996). Osteopontin stimulates gelsolin-associated phosphoinositide levels and phosphatidylinositol triphosphate-hydroxyl kinase.. Mol Biol Cell.

[31] Chellaiah M, Fitzgerald C, Alvarez U, Hruska K (1998). c-Src is required for stimulation of gelsolin-associated phosphatidylinositol 3-kinase.. J Biol Chem.

[32] Schneider A, Younis RH, Gutkind JS (2008). Hypoxia-induced energy stress inhibits the mTOR pathway by activating an AMPK/REDD1 signaling axis in head and neck squamous cell carcinoma.. Neoplasia.

[33] Cho Y, Lee HW, Kang HG, Kim HY, Kim SJ (2015). Cleaved CD44 intracellular domain supports activation of stemness factors and promotes tumorigenesis of breast cancer.. Oncotarget.

[34] Cichy J, Puré E (2003). The liberation of CD44.. J Cell Biol.

[35] Okamoto I, Tsuiki H, Kenyon LC, Godwin AK, Emlet DR (2002). Proteolytic cleavage of the CD44 adhesion molecule in multiple human tumors.. Am J Pathol.

[36] Liu X, Rose DP (1994). Stimulation of type IV collagenase expression by linoleic acid in a metastatic human breast cancer cell line.. Cancer Letters.

[37] Masson D, Denis MG, Denis M, Blanchard D, Loirat MJ (1999). Soluble CD44: quantification and molecular repartition in plasma of patients with colorectal cancer.. Br J Cancer.

[38] Gupta A, Cao W, Sadashivaiah K, Chen W, Schneider A (2013). Promising noninvasive cellular phenotype in prostate cancer cells knockdown of matrix metalloproteinase 9.. ScientificWorldJournal.

[39] Pratap J, Lian JB, Javed A, Barnes GL, van Wijnen AJ (2006). Regulatory roles of Runx2 in metastatic tumor and cancer cell interactions with bone.. Cancer Metastasis Rev.

[40] Pratap J, Javed A, Languino LR, van Wijnen AJ, Stein JL (2005). The Runx2 osteogenic transcription factor regulates matrix metalloproteinase 9 in bone metastatic cancer cells and controls cell invasion.. Mol Cell Biol.

