# Supplementary material for: Identification of sequence-specific interactions of the CD44-intracellular domain with RUNX2 in the transcription of matrix metalloprotease-9 in human prostate cancer cells
Source: Cancer Drug Resist. Author manuscript; Available in PMC 2020 Oct 14. (PMC7556329; doi:10.20517/cdr.2020.21)
Supplement: Supplementary file 1 [file NIHMS1622835-supplement-supplementary_materials.pdf]

Figure S1

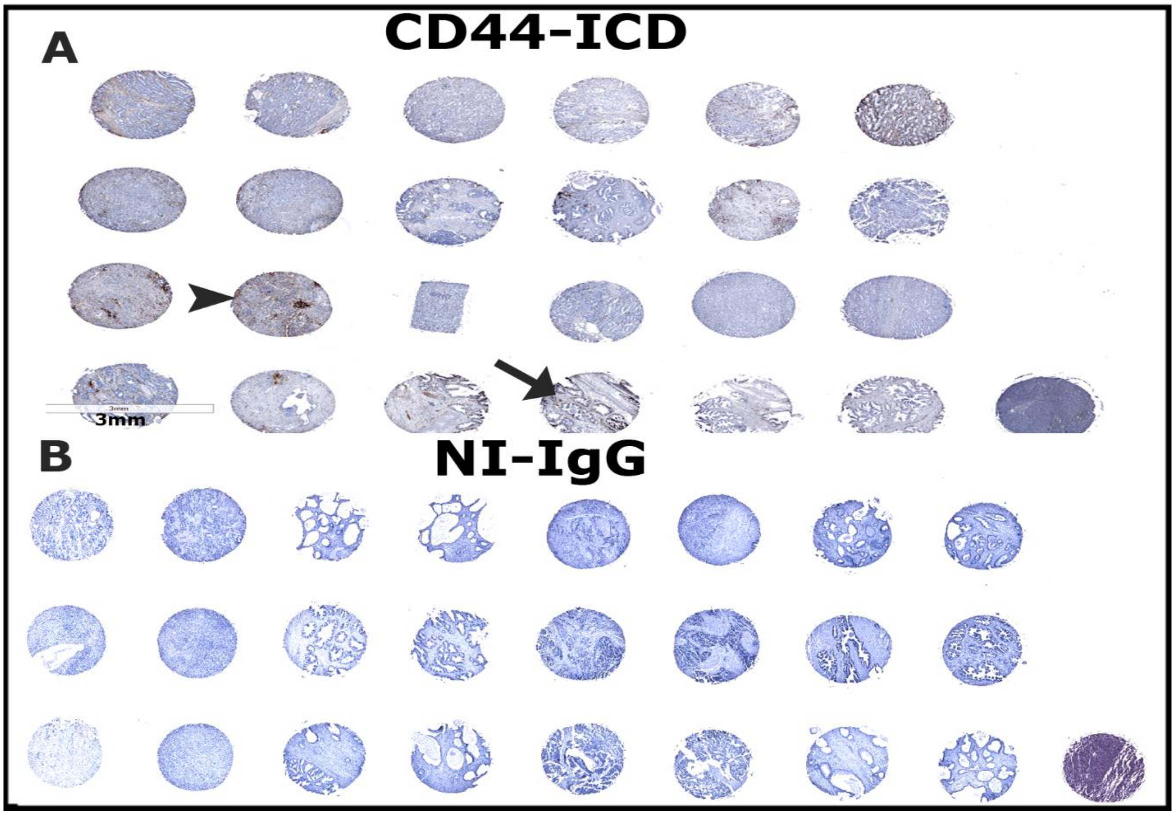

Figure S1: Immunohistochemistry analysis of TMA in adjacent normal prostate tissue and adenocarcinoma (stage IV). A-B Lower magnification view of tissue microarray (TMA) of normal prostate tissue and different stages of adenocarcinoma (prostate cancer) (top panel). TMA sections containing 24 cases were stained with an antibody to CD44-ICD **(A)** and species-specific non-immune serum (NI-IgG) **(B).** Then sections were scanned in using an Aperio Scanscope® CS instrument (Aperio Scanscope CS system, Vista, CA). Staining was repeated two times.

Figure S2

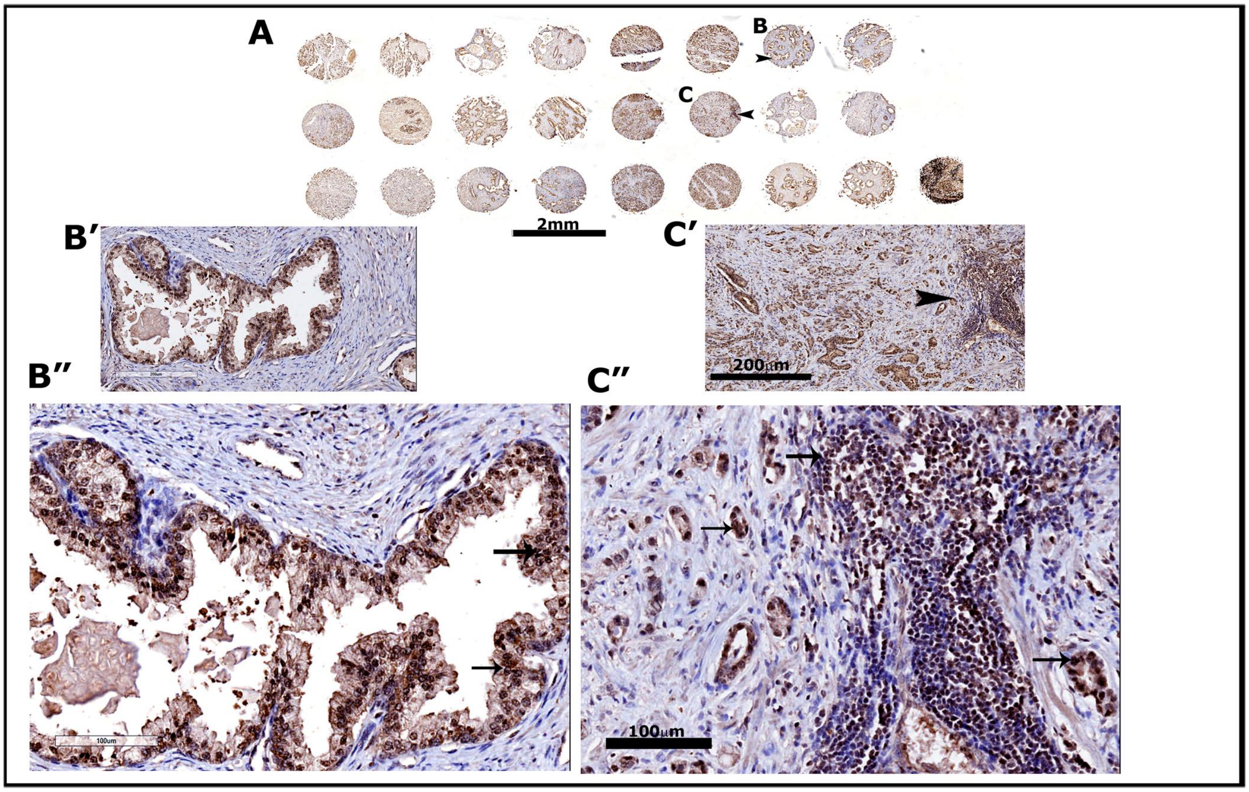

Figure S2: Immunohistochemistry analysis of TMA in adjacent normal prostate tissue and adenocarcinoma (stage IV). A-C Lower magnification view of tissue microarray (TMA) of normal prostate tissue and different stages of adenocarcinoma (prostate cancer). **(A)** TMA sections containing 24 cases were stained with an antibody to CD44. **B and C** in CD44 panel (A) represent normal prostatic and adenocarcinoma (stage IV) tissue sections, respectively. Arrowheads in B and C represent area of magnification in B' and C'. Arrow in C'' represents the magnified region of C' and arrowhead in B" and C" represents nuclear localization of CD44. Sections were scanned using an Aperio Scanscope® CS instrument (Aperio Scanscope CS system, Vista, CA). The staining was repeated two times.

Figure S3

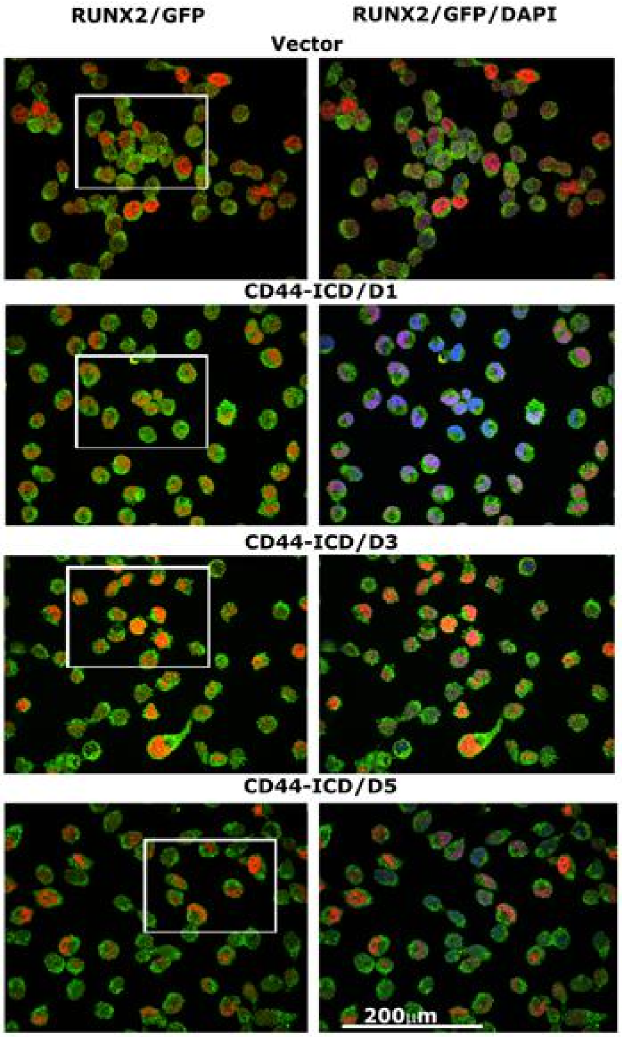

Figure S3: Confocal microscopy analysis of the specificity of interaction of RUNX2/CD44-ICD deletion constructs. Confocal microscopy analyses of PC3 cells expressing indicated constructs and vector are shown. Cells were stained with antibodies to GFP (green) and RUNX2 (red). Cells were counterstained with DAPI for nuclear staining (blue). Magnification – 200μm
